# Clinical characteristics of hospitalized term and preterm infants with community-acquired viral pneumonia

**DOI:** 10.1186/s12887-022-03508-7

**Published:** 2022-07-27

**Authors:** Xinxian Guan, Shasha Gao, He Zhao, Huiting Zhou, Yan Yang, Shenglin Yu, Jian Wang

**Affiliations:** 1 Department of Neonatology, Children’s Hospital of Soochow University, Suzhou, China; 2 Institute of Pediatric Research, Children’s Hospital of Soochow University, Suzhou, China

**Keywords:** Newborn, Pneumonia, Virus, Preterm, Coinfection

## Abstract

**Background:**

Pneumonia is a serious problem that threatens the health of newborns. This study aimed to investigate the clinical characteristics of hospitalized term and preterm infants with community-acquired viral pneumonia.

**Methods:**

This was a retrospective analysis of cases of community-acquired viral pneumonia in the Neonatal Department. Nasopharyngeal aspirate (NPA) samples were collected for pathogen detection, and clinical data were collected. We analysed pathogenic species and clinical characteristics among these infants.

**Results:**

RSV is the main virus in term infants, and parainfluenza virus (PIV) 3 is the main virus in preterm infants. Patients infected with PIV3 were more susceptible to coinfection with bacteria than those with respiratory syncytial virus (RSV) infection (*p* < 0.05). Preterm infants infected with PIV3 were more likely to be coinfected with bacteria than term infants (*p* < 0.05), mainly gram-negative bacteria (especially Klebsiella pneumonia). Term infants with bacterial infection were more prone to fever, cyanosis, moist rales, three concave signs, elevated C-reactive protein (CRP) levels, respiratory failure and the need for higher level of oxygen support and mechanical ventilation than those with simple viral infection (*p* < 0.05). The incidence of hyponatremia in neonatal community-acquired pneumonia (CAP) was high.

**Conclusions:**

RSV and PIV3 were the leading causes of neonatal viral CAP. PIV3 infection is the main cause of viral CAP in preterm infants, and these individuals are more likely to be coinfected with bacteria than term infants, mainly gram-negative bacteria. Term infants with CAP coinfected with bacteria were more likely to have greater disease severity than those with single viral infections.

## Introduction

Pneumonia is one of the most common infectious diseases in the neonatal period and accounts for 46% of all neonatal diseases [1]. Moreover, the mortality rate of pneumonia is 1.2%, which ranks highest among all neonatal infectious diseases; thus, pneumonia is a serious problem that threatens the health of newborns [2]. The main pathogens of neonatal pneumonia are bacteria, viruses, and fungi [3]. In recent years, many studies of bacterial pneumonia in neonates have been published [4], but information on viral pneumonia in neonates is limited. Many viruses can damage the airway epithelial layer, thus increasing the likelihood of both adherence to the respiratory tract and bacterial translocation, two of the critical first steps in causing infection [5]. Viruses can also lead to dysfunction of the immune system, thereby promoting bacterial infection [6]. We retrospectively analysed all preterm and term neonates with community-acquired viral pneumonia over a 5-year period to study the aetiology and clinical features of these infants.

## Materials and methods

### Patients

The present study was a retrospective analysis of newborn patients with community-acquired viral pneumonia. The general clinical data, clinical signs and symptoms, auxiliary examination results, complications and prognoses were collected and analysed from the hospital medical records system.

The inclusion criteria were as follows: the patients were hospitalized in the Neonatal Department of Children’s Hospital of Soochow University (Suzhou, China) between January 2017 and December 2021 and were diagnosed with community-acquired pneumonia (CAP); and the aetiology of these cases must be positive for respiratory syncytial virus (RSV), adenovirus, influenza virus (Inf) A, Inf B, or parainfluenza virus (PIV1, PIV2, and PIV3).

The exclusion criteria were as follows: patients with incomplete clinical data and severe basic diseases, such as congenital heart disease, congenital immunodeficiencies and incomplete medical data [7, 8].

### Diagnostic criteria and data collection

CAP refers to clinical signs and symptoms of pneumonia acquired outside a hospital setting [9] and is diagnosed based on clinical findings (fever, cough, and difficulty in breathing), physical examination findings (tachypnoea, chest retraction, and decreased breath sounds or rales), and radiological findings [10]. Chest radiographs were evaluated by a radiologist trained in reading and interpreting radiographs according to the World Health Organization’s (WHO) guidelines [11]. Respiratory failure is defined as the failure to maintain either normal delivery of oxygen to the tissues or normal removal of carbon dioxide from the tissues. Respiratory failure occurs when there is an imbalance between the respiratory workload and ventilatory strength and endurance. The suggested cutoffs for diagnosing respiratory failure include two or more of the following: PaCO2 > 60 mmHg, PaO2 < 50 mmHg or O2 saturation < 80% with an FiO2 of 1.0 and pH < 7.25 [12]. Heart failure (HF) is defined as the failure of the heart when it supplies blood to either systemic or pulmonary circulation at an appropriate rate of flow, or to receive venous return at an appropriate filling pressure, but produces adverse effects on the heart, the circulation, and the patient [13]. The diagnosis and treatment of HF are based on Canadian Cardiovascular Society Guidelines [13]. Pulmonary air leak syndrome (PALS) comprises several different clinical conditions, such as pulmonary interstitial emphysema (PIE), pneumomediastinum, pneumothorax and pneumopericardium, which all results from alveolar over distension and air leakage outside the lungs [14]. In this study, PALS is comprised of pneumothorax and pneumomediastinum. The treatment of mechanical ventilation (MV) in this research includes invasive MV and non-invasive ventilation (NIV), and invasive MV includes high frequency oscillatory ventilation (HFOV) and conventional mechanical ventilation (CMV).

### Pathogen testing

Nasopharyngeal aspirates (NPA) were collected under strict aseptic operations within 24 h from all hospitalized patients (*n* = 375) to identify the pathogen. The samples were divided into three subsamples for pathogen detection. One subsample was used to detect seven common respiratory viruses as previously described by direct immunofluorescence analysis, and the remaining two subsamples were used to detect and identify bacteria using bacterial culture and *Mycoplasma* by using PCR analysis. The study period overlapped with coronavirus disease 2019 (COVID-19) pandemic, COVID-19 real-time polymerase chain reaction (RT-PCR) test was performed by nasopharyngeal swab method for the infants from March 2020 to December 2021(*n* = 89).

#### Immunofluorescence analysis for respiratory virus pathogen detection

A total of 1–2 mL of NPA was mixed with PBS and centrifuged at (400–600) × g for 15 min. Then, the supernatant was discarded, and the remaining sample was washed three times with PBS (5 mL). PBS (0.5 to 1 mL) was added after centrifugation to make the cell suspension. Subsequently, seven wells (25 μL/well) were spotted on the slide, air-dried at room temperature, and fixed in cold acetone for 10 min. Then, 20 μL of immunofluorescent reagent (containing fluorescein-FITC-labelled monoclonal antibody) was added to each of the wells. After incubation at 37 °C for 30 min and rinsing 3 times with PBS, the glycerol buffer solution was air-dried and ready for further analysis. Seven common respiratory viruses (including RSV, adenovirus, InfA, Inf B, PIV1, PIV2, and PIV3) were detected by direct immunofluorescence analysis. A positive negative control is provided by the kit (D3 Ultra DFA respiratory virus screening and identification kit, Athens, Ohio, USA). Bright yellow–green fluorescence and/or fluorescent spots in the nucleus/cytoplasm were considered positive.

#### RT-PCR for COVID-19

Laboratory confirmation of the Severe Acute Respiratory Syndrome Coronavirus 2 (SARS-CoV-2) RNA was performed by RT-PCR. Briefly, according to the manufacturer’s instruction, we extracted the nucleic acid from nasopharyngeal swab samples by using the Viral Nucleic Acid Kit (Health, Ningbo, China). We also used COVID-19 detection kits (Bioperfectus, Taizhou, China) in detecting the ORF1ab and the N genes. At the same time, we adopted the procedure of RT-PCR assay from the WHO protocol (2020b). A positive test was defined as a cycle threshold value (Ct value) less than 35 and laboratory confirmation of COVID-19 was based on the positive results for both ORF1ab and the N genes.

#### Bacterial culture for bacterial detection

Bacteria were tested by inoculating NPA samples on blood plates that were read after incubating for 18–20 h. If bacterial growth was > 10^4^ colony forming units/mL, it was considered significant. Morphology selection depended on experienced clinical laboratory physicians.

#### PCRs for Mycoplasma

*Mycoplasma* pneumoniae were detected by PCR. NPA samples were centrifuged at 12,000 × g for 5 min. DNA was obtained from the NPA samples (200 μL) using DNA-EZ Reagents (Sangon Biotech, Shanghai, China) in accordance with the manufacturer’s instructions. A final volume of 100 μL containing DNA was eluted for detection of *Mycoplasma* pneumoniae gene amplification via real-time PCR.

### Statistical analysis

Statistical analysis was performed using SPSS v.17.0 for Windows (SPSS Inc., Chicago, IL). Normally distributed data are expressed as the mean ± standard deviation, and nonnormally distributed data are expressed as the median and interquartile range. Normally distributed data were compared using the independent samples t test, and nonnormally distributed data were compared using the Kruskal–Wallis test. Categorical data are presented as numbers and percentages. The chi-square and Fisher exact tests were used to compare categorical data. All tests were two-tailed, and *P* < 0.05 was considered statistically significant.

## Results

Three hundred seventy-five newborns were enrolled and retrospectively analysed in this study. No deaths were reported. There were 248 males and 127 females. Of the 375 patients, 344 were term infants, and 31 were preterm infants. All term infants were younger than 28 days old at admission. The postmenstrual age (PMA) of all preterm infants was less than 44 weeks after birth.

### Comparison of pathogens in preterm and term infants

Of the 375 community-acquired viral pneumonia cases, 140 patients were coinfected with bacteria, 3 patients were coinfected with mycoplasma. The types of bacteria included *Staphylococcus aureus* (*n* = 42), *Escherichia coli* (*n* = 32), *Klebsiella pneumoniae* (*n* = 22), *Streptococcus viridans* (*n* = 20), *Moraxella catarrhalis* (*n* = 13), *Aerobacter cloacae* (*n* = 5), *Enterobacter aerogenes* (*n* = 3)*, Haemophilus influenzae* (*n* = 2) and *Proteus mirabilis* (*n* = 1). All admitted infants through the pandemic were tested negative for COVID-19.

Full-term infants were more likely to be infected with RSV than preterm infants (*p* < 0.001). Preterm infants were more likely to be infected with PIV3 than term infants (*p* < 0.001). In addition, preterm infants were more likely to be coinfected with bacteria than term infants (*p* < 0.001), especially Gram-negative bacteria (*p* < 0.001), such as *Klebsiella pneumoniae* (*p* < 0.001) (Table 1).

**Table 1:** Comparison of pathogens in preterm and term infants

Clinical Parameters	Term infants *n* = 344	Preterm infants *n* = 31	*P value*
Demographic data			
Sex (male, %)	224(65.1)	24(77.4)	*0.166*
Age at admission (median, IQR, days)	19.0(13.3–24.0)	28(26–40)	< *0.001*
PMA (mean ± std, weeks)	41.7 ± 1.5	37.8 ± 2.3	< *0.001*
Virus type			
RSV (n, %)	308(89.5)	16(51.6)	< *0.001*
PIV3(n, %)	24(7.0)	11(35.5)	< *0.001*
Other viruses (n, %)	12(3.5)	3(9.7)	*0.228*
RSV and PIV3 coinfection (n, %)	1(0.3)	0(0.0)	*0.999*
Co-infection distribution			
Co-infection with bacteria (total, n, %)	117(34.0)	23(74.2)	< *0.001*
Gram positive bacteria (n, %)	56(16.3)	6(19.4)	*0.659*
Staphylococcus aureus (n, %)	39(11.3)	3(9.7)	*0.999*
Streptococcus viridans (n, %)	17(4.9)	3(9.7)	*0.480*
Gram negative bacteria (n, %)	61(17.7)	17(54.8)	< *0.001*
Escherichia coli (n, %)	26(7.6)	6(19.4)	*0.055*
Klebsiella pneumoniae (n, %)	14(4.1)	8(25.8)	< *0.001*
Moraxella catarrhalis (n, %)	12(3.5)	1(3.2)	*0.999*
Aerobacter cloacae (n, %)	5(1.5)	0(0)	*0.999*
Enterobacter aerogenes (n, %)	2(0.6)	1(3.2)	*0.596*
Haemophilus influenzae (n, %)	2(0.6)	0(0)	*0.999*
Proteus mirabilis (n, %)	0(0)	1(3.2)	*0.129*
Mycoplasma (n, %)	2(0.6)	1	*0.596*

*PMA* postmenstrual age, *RSV* respiratory syncytial virus, *PIV* parainfluenza virus

In all cases of community-acquired viral pneumonia, regardless of the viral infection, newborns with coinfection, especially bacterial infection, were more prone to respiratory failure (43/140). The probability of respiratory failure in children with simple virus infection (30/232) was lower (χ2 = 17.51, *p* < 0.001) than in those coinfected with bacteria (Table 2).

**Table 2:** Comparison of clinical characteristics of term infants and premature infants with viral pneumonia based on coinfection with bacteria

Clinical Parameters	Term infants (*n* = 342)			Preterm infants (*n* = 30)		
	Co-infection with bacteria(*n* = 117)	Simple virus infection(*n* = 225)	*P* value	Co-infection with bacteria(*n* = 23)	Simple virus infection(*n* = 7)	*P* value
**Demographic data**						
** Gestational age** (mean ± std, weeks)	39.2 ± 1.4	38.9 ± 1.2	*0.058*	32.5 ± 2.4	31.7 ± 2.0	*0.441*
** Sex (male, %)**	74(63.2)	148(65.8)	*0.642*	18(78.3)	6(85.7)	*0.999*
** Birth weight** (mean ± std, g)	3279 ± 409	3295 ± 359	*0.709*	1911 ± 421	1890 ± 445	*0.908*
** Age at admission** (median, IQR, days)	20(13–24)	19(13.5–24)	*0.579*	28(25–36)	31(26–60)	*0.631*
** PMA** (mean ± std, weeks)	——	——	——	37.8 ± 2.4	37.9 ± 2.2	*0.965*
**Clinical symptoms**						
** Fever (n, %)**	32(27.4)	30(13.3)	*0.001*	2(8.7)	1(14.3)	*0.999*
** Cough (n, %)**	112(95.7)	223(99.1)	*0.090*	23(100)	7(100)	*0.999*
** Apnoea (n, %)**	7(6.0)	8(3.6)	*0.298*	9(39.1)	1(14.3)	*0.372*
** Cyanosis (n, %)**	18(15.4)	9(4.0)	< *0.001*	10(43.5)	2(28.6)	*0.669*
** Tachypnoea (n, %)**	74(63.2)	125(55.6)	*0.171*	19(82.6)	6(85.7)	*0.999*
** Refusal to feed (n, %)**	23(19.7)	56(24.9)	*0.276*	9(39.1)	2(28.6)	*0.999*
** Vomit/diarrhoea (n, %)**	4(3.4)	20(8.9)	*0.060*	4(17.4)	2(28.6)	*0.603*
**Physical examination**						
** Moist rales (n, %)**	68(58.1)	155(68.9)	*0.047*	21(91.3)	4(57.1)	*0.068*
** Wheezing rales (n, %)**	36(30.8)	68(30.2)	*0.917*	9(39.1)	0(0)	*0.071*
** Three concave sign (n, %)**	60(51.3)	59(26.2)	< *0.001*	12(52.2)	6(85.7)	*0.193*
**Laboratory tests**						
** WBC** (mean ± std, × 10^9^/L)	8.88 ± 3.02	8.57 ± 2.75	*0.327*	11.24 ± 4.75	9.38 ± 3.29	*0.344*
** Proportion of neutrophils to WBC** (median, IQR, %)	26.70(19.20–34.45)	26.70(20.80–33.60)	*0.617*	26.00(22.60–31.40)	55.70(28.90–63.80)	*0.001*
** CRP **(median, IQR, mg/L)	2.49(0.77–6.95)	0.38(0.08–1.33)	< *0.001*	1.59(0.52–9.87)	1.11(0.05–5.33)	*0.311*
**Complication**						
** Respiratory failure (n, %)**	31(26.5)	25(11.1)	< *0.001*	12(52.2)	5(71.4)	*0.427*
** Supplemental oxygen (n, %)**	29(24.8)	30(13.3)	*0.008*	9(39.1)	3(42.9)	*0.999*
** MV (n, %)**	4(3.4)	0(0)	*0.024*	5(21.7)	2(28.6)	*0.999*
HFOV (n, %)	1(0.9)	0(0)	*0.342*	0(0)	0(0)	——
CMV (n, %)	0(0)	0(0)	——	2(8.7)	1(14.3)	*0.999*
NIV (n, %)	3(2.6)	0(0)	*0.072*	3(13.0)	1(14.3)	*0.999*
** HF (n, %)**	6(5.1)	3(1.3)	*0.085*	3(13.0)	2(28.6)	*0.565*
Diuretic agents (n, %)	6(5.1)	3(1.3)	*0.085*	3(13.0)	2(28.6)	*0.565*
Inotropic agents (n, %)	6(5.1)	3(1.3)	*0.085*	3(13.0)	2(28.6)	*0.565*
Vasodilator agents (n, %)	5(4.3)	2(0.9)	*0.090*	3(13.0)	1(14.3)	*0.999*
** PALS (n, %)**	3(2.6)	3(1.3)	*0.698*	2(8.7)	0(0)	*0.999*
Pneumothorax (n, %)	3(2.6)	2(0.9)	*0.453*	2(8.7)	0(0)	*0.999*
Pneumomediastinum (n, %)	2(1.7)	1(0.4)	*0.563*	0(0)	0(0)	——
** Hyponatremia (n, %)**	35(29.9)	63(28.0)	*0.710*	14(60.9)	4(57.1)	*0.999*
** Duration of hospital stay** (median, IQR, days)	11(9–13)	11(9–13)	*0.692*	22.13 ± 11.01	12.86 ± 7.20	*0.046*

*PMA* postmenstrual age, *CRP* C-reactive protein, *WBC* white blood cell, *MV* mechanical ventilation**,** *HFOV* invasive high frequency oscillatory ventilation, *CMV* invasive conventional mechanical ventilation, *NIV* non-invasive ventilation, *HF* heart failure, *PALS* pulmonary air leak syndrome

### The distribution of virus species and monthly distribution of respiratory virus detection

**Fig. 1:**
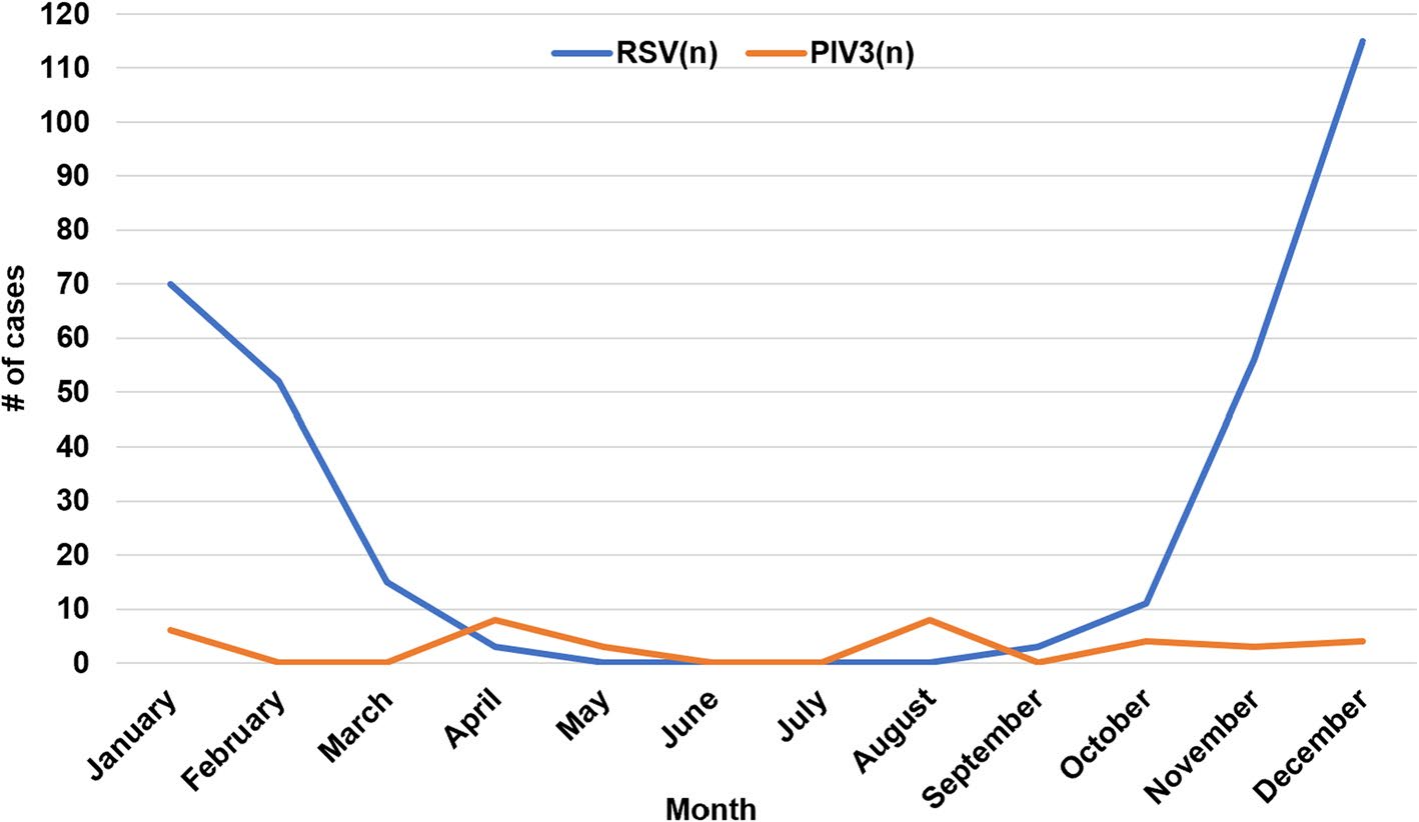
Monthly distribution of CAP in neonates with RSV and PIV3 infection

Among the 375 enrolled patients, 322 were infected with RSV alone (85.9%), 2 were infected with both RSV and Inf A (0.5%), 1 was infected with both RSV and PIV3 (0.3%), 35 were infected with PIV3 alone (9.3%), 10 were infected with Inf A alone (2.7%) and 5 were infected with Inf B alone (1.3%). No patient was infected with adenovirus, PIV1 or PIV2. RSV infection mainly occurred in January, February, November and December, which showed obvious seasonal prevalence. However, PIV3 infection did not show significant seasonal prevalence (Fig. 1).

### Patients infected with PIV3 were more prone to co-infection with bacteria than those with RSV infection. Preterm infants were more susceptible to co-infection with bacteria than term infants

**Fig. 2:**
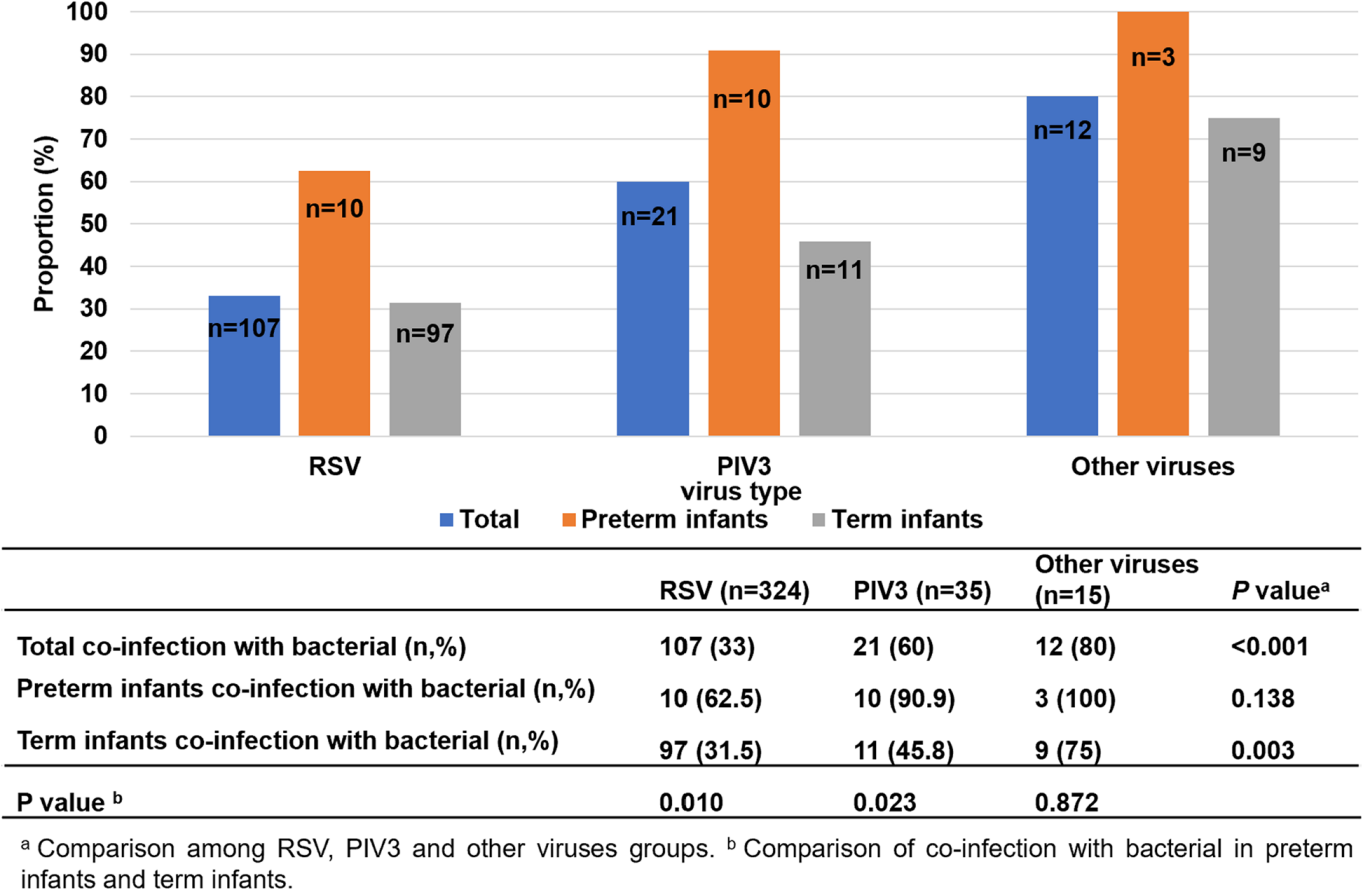
The proportion of coinfection with bacteria of different viruses in preterm and term infants

In all children with community-acquired viral pneumonia, their combined bacterial infection was compared. One child with mixed infection of RSV and PIV3 was excluded because he had no bacterial infection. The prevalence of RSV pneumonia complicated with bacterial infection was 33.0% (107/324), and the prevalence of PIV3 pneumonia complicated with bacterial infection was 60% (21/35). Because the total number of children with other types of viral (Inf A and Inf B) pneumonia was small, statistical analysis was carried out together, and the prevalence of combined bacterial infection was 80% (12/15). Statistical analysis showed that patients infected with PIV3 and other viruses were more likely to be coinfected with bacteria than patients infected with RSV (*p* < 0.05). Among RSV-infected patients, preterm infants were more likely to be complicated (*p* < 0.01) with bacterial infection than term infants. The same trend was found in PIV 3-infected infants (*p* < 0.05) (Fig. 2**)**.

### Clinical characteristics of coinfected with bacteria in term infants and premature infants with viral pneumonia

Of the 375 patients with community-acquired viral pneumonia, two cases of coinfection with mycoplasma in term infants and one case of coinfection with mycoplasma in preterm infants were excluded from the analysis. Therefore, the clinical features of coinfection with bacteria among term infants (342 cases) and premature infants (30 cases) with viral pneumonia are summarized in Table 2. The percentage of coinfection with bacteria was different between term and preterm infants (*p* < 0.05). There were 117 term infants coinfected with bacteria (117/342), and there were 23 preterm infants coinfected with bacteria (23/30). Our results suggested that premature infants are more likely to be complicated with bacterial infection.

For laboratory tests, the white blood cell (WBC) count was not affected by coinfection with bacteria in term or preterm infants. The proportion of neutrophils to WBCs was not different between term infants with coinfection with bacteria and those with simple virus infection. However, in preterm infants, the prevalence of neutrophils to WBCs was different between those with coinfection with bacteria and those with simple virus infection. The level of C-reactive protein (CRP) in term infants was different between those with coinfection with bacteria and those with simple virus infection. The level of CRP was not affected in preterm infants with co-infection with bacteria and those with simple virus infection.

In the term infant group, children with bacterial infection are more likely to have fever and cyanosis symptoms than children with simple virus infection. The three concave signs are more obvious among term infants with bacterial infection, their CRP values are higher (*p* < 0.01), they are more likely to be complicated with respiratory failure, and they are more likely to need oxygen support and mechanical ventilation treatment, including invasive and non-invasive mechanical ventilation. The signs of pulmonary moist rales in children with simple virus infection were significantly more common than those in children with bacterial infection.

In the preterm infant group, the hospitalization time of infants with bacterial infection was longer than that of infants with simple virus infection, and the prevalence of neutrophils to WBC was lower (*p* < 0.014) than that of infants with simple virus infection.

## Discussion

Neonatal immune dysfunction, as well as neonatal lung development, is not fully developed and vulnerable to the invasion of external pathogens [15]. Pathogens can be transmitted to newborns through droplets and contact [16]. Because of the complexity of the community environment and the variation in regions and seasons, the distribution of pathogens also differs. In addition, the incidence of disease increases rapidly, which is the focus of current research [17].

In our study, the peak incidence of RSV pneumonia occurred during the winter and early spring. Similar results were reported in our previous study with a subtropical climate [18, 19], and the peak of RSV activity was in the winter and spring seasons [19].

In Suzhou, China, the infection rate of COVID-19 was low, epidemic data is obtained from the Sina Real-Time Epidemic website (https://news.sina.cn/zt_d/yiqing0121) [20]. We had tested hospitalized children for SARS-CoV-2 and there were no positive cases during study period. The current research showed that RSV and PIV3 were the major viral pathogens in neonatal community-acquired viral pneumonia, especially RSV. Few patients were infected with InfA or InfB. RSV was the main virus in term newborns. PIV3 was the main virus in preterm infants. Unfortunately, there are currently no vaccines available against pathogens (RSV, PIVs, influenza virus) for infants under 6 months of age [21]. In newborns, especially in preterm infants, palivizumab is the only licenced treatment to help reduce the burden of RSV [22]. Unfortunately, palivizumab use is limited in the Suzhou area. Additionally, Preterm infants are more likely to co-infect with bacteria than term infants, especially gram-negative bacteria, such as *Klebsiella pneumoniae*. Therefore, in clinical work, we can preliminarily distinguish potential pathogens for newborns with CAP based on whether they are preterm or term infants and thus select the targeted treatment schemes.

In the present study, patients with PIV3 infection were more likely to be infected with bacteria than patients with RSV infection (60.0% vs. 33.0%). Additionally, patients with other virus (InfA, InfB) infections were more likely to be infected with bacteria than patients with RSV infections (80.0% vs. 33.0%). Among RSV-infected patients, preterm infants were more likely to be complicated with bacterial infection than term infants, and the same trend was found in PIV3-infected infants. This result suggested that preterm infants are more susceptible to coinfection with bacteria than term infants. Therefore, in the clinic, when treating patients with PIV3 and other virus (InfA, InfB) infections or preterm infants, we should be alert to the possibility of combined bacterial infection and relax the indications for the use of antibiotics on the basis of support and symptomatic treatment. For patients with RSV infection, antibiotics should be used as soon as a concurrent bacterial infection is detected.

Not everyone with neonatal viral pneumonia will have prodromal symptoms, as the incidence of these symptoms usually varies between 30 and 65% depending on the pathogen [23]. In this study, patients with mild infections only showed symptoms of mild cough and low fever, while patients with severe infections had serious cough, high fever, apnoea, cyanosis, tachypnoea, refusal to feed, vomit or diarrhoea, three concave signs, increased moist rales, wheezing in the lungs, and complications with respiratory failure, HF and PALS.

This study also showed that preterm infants are more susceptible to coinfection, especially for bacteria, which is related to the lower immune function of preterm infants than that of term infants [24, 25]. The trachea of premature infants is narrow, and the wall of the trachea easily collapses. The abundant capillaries and weak ciliary movement function provide a good environment for the attachment and reproduction of pathogenic bacteria [26]. Because the immune system of premature infants is not fully developed, their immune function is low [27, 28]. Neonatal immune function, especially that of the local airway, is also underdeveloped with lower levels of secretory IgA, which serves an anti-infectious role [29]. Foetal immunoglobulins are mostly transmitted from the mother, but this physiological process mainly occurs in the middle and late stages of pregnancy. The IgG level of full-term newborns can reach the maternal level [30]. Respiratory virus infection is often accompanied by bacterial infection [31]. Because the humoral and cellular immunity of premature infants at small gestational age are at a low level [24], the damaged respiratory mucosa and inhibited immune function by respiratory viruses induce the risk of bacterial infection. Therefore, newborns are more easily infected by a variety of pathogens. Therefore, targeted measures, such as perinatal detection and health care, should be taken to reduce the birth of premature infants. When pneumonia occurs in premature infants, corresponding treatment measures, including respiratory support, immune support, enteral nutrition, parenteral nutrition support and sodium supplements, should be actively adopted.

Hyponatremia is relatively common in pneumonia, with one large Italian series reporting a rate of 45% [32]. In our study, the overall incidence of hyponatremia was 30.9% (116/375); however, hyponatremia was mild in the majority of cases, from 130 mmol/l to 135 mmol/l. Studies in developing countries have shown this to be associated with increasing severity of pneumonia and risk of death. Factors increasing the risk of dehydration and potentially hyponatremia include reduced nutrient/water intake and increased evaporative losses as a result of both increased respiratory rate and increased core temperature. Sometimes it may be a result of additional losses from vomiting and diarrhoea. Pneumonia is widely cited as a potential cause of syndrome of inappropriate antidiuretic hormone secretion (SIADH) [33], but no studies have examined the biochemistry and pathophysiology of hyponatremia in children with pneumonia with sufficient rigor to be able to differentiate adequately between SIADH and salt depletion; therefore, hyponatremia has frequently been ascribed to SIADH. Some scholars suspect that hyponatremia principally occurs secondarily to dehydration in most children considered to have SIADH. This has major implications for acute patient management, as SIADH is managed with fluid restriction, and dehydration clearly requires rapid volume replacement. Further studies are urgently required to address this question in the future.

In all patients involved in this study, the main pathogens that were co-infected were *Staphylococcus aureus*, *Escherichia coli*, *Klebsiella pneumoniae* and *Streptococcus viridans*, which was similar to other reports [34]. In addition, this article also found that in term infants with concurrent bacterial infections, the symptoms are more severe and more likely to have respiratory failure. Some of these patients require not only oxygen support but also NIV or invasive MV. Therefore, term infants with viral and bacterial infections are more severe than those with pure viral infections [35, 36], and more attention should be devoted to respiratory management [37, 38] and supportive care. Once the results of NPA culture are confirmed (before those of drug sensitivity testing are clear), appropriate antibiotics for common bacteria can be empirically selected. After the report of bacterial drug sensitivity tests, the type of antibiotics should be adjusted according to the treatment effect.

Neutrophils play an important and active role in the body's nonspecific immunity. In the present study, the total number of leukocytes in preterm infants combined with bacterial infection was numerically higher than that in preterm patients with simple virus infection; although the difference was not statistically significant, the prevalence of neutrophils to WBC was lower than that in patients with simple virus infection (*p* < 0.05). The hospital stay in preterm infants with bacterial infection is also longer than that in preterm patients with simple virus infection. When bacteria and other microbial pathogens invade and inflammatory reactions occur, they can reach the inflammatory site under the influence of chemokines, devour bacteria and tissue fragments, and prevent the diffusion of pathogenic microorganisms in the body [39]. When the inflammatory reaction is strong, a large number of neutrophils stored in bone marrow are released into the blood, and the level of neutrophils increases. However, neutrophils were depleted in bone marrow when the infection was very serious, resulting in a decrease in neutrophil expression levels [40]. Therefore, when the prevalence of neutrophils to WBC is reduced in preterm infants, the risk of bacterial infection may increase, resulting in a prolonged hospital stay.

## Conclusion

RSV and PIV3 are the main pathogens of neonatal viral pneumonia. It is necessary to consider the possibility of RSV infection in neonates with viral CAP in winter and early spring. In PIV3-infected newborns and premature infants, we should be alert to the possibility of combined bacterial infection. Preterm infants are more prone to PIV3 infection and coinfection with bacteria. Term infants are more prone to be infected with RSV. Term infants with bacterial infection are more prone to respiratory failure than simple virus infection. Patients with respiratory failure should be closely monitored and supported in a timely manner by inhaling oxygen and ventilation. When the prevalence of neutrophils to WBCs in preterm infants is reduced, it is easily complicated by bacterial infection. Additionally, hyponatremia cannot be ignored in children with pneumonia.

However, this study also has some shortcomings: the number of patients involved, especially preterm infants, was small. In the future, we will conduct more joint research with hospitals and communities in multiple regions. We will expand the inclusion criteria and include some children with insignificant performance in the study.

## Data Availability

The datasets generated and/or analysed during the current study are not publicly available due to the datasets need to be kept confidential, but they are available from the corresponding author upon reasonable request.

## Abbreviations

NPA: Nasopharyngeal aspirates
CAP: Community-acquired pneumonia
RSV: Respiratory syncytial virus
PIV: Parainfluenza virus
Inf: Influenza virus
CRP: C-reactive protein
WHO: World Health Organization
PMA: Postmenstrual age
SIADH: Syndrome of inappropriate antidiuretic hormone secretion
RT-PCR: Real-time polymerase chain reaction
SARS-CoV-2: Severe Acute Respiratory Syndrome Coronavirus 2
COVID-19: Coronavirus disease 2019
HF: Heart failure; PALS: pulmonary air leak syndrome
PIE: Pulmonary interstitial emphysema
MV: Mechanical ventilation
HFOV: High frequency oscillatory ventilation
CMV: Conventional mechanical ventilation
NIV: Non-invasive ventilation
